# The effects of normal vaginal birth simulation training on the clinical skills of midwifery students: a quasi-experiment study

**DOI:** 10.1186/s12909-023-04319-9

**Published:** 2023-05-19

**Authors:** Zahra Sadat Pajohideh, Solmaz Mohammadi, Fatemeh Keshmiri, Azam Jahangirimehr, Azam Honarmandpour

**Affiliations:** 1 MSc of Midwifery, Department of Midwifery, Shoushtar Faculty of Medical Sciences, Shoushtar, Iran; 2 Reproductive Health Promotion Research Center, Ahvaz Jundishapur University of Medical Sciences, Ahvaz, Iran; 3 Medical Education Department, Educational Developmental Center, Shahid Sadoughi University of Medical Sciences, Yazd, Iran; 4 MSc of Biostatistics, Shoushtar Faculty of Medical Sciences, Shoushtar, Iran

**Keywords:** Simulation training, Vaginal delivery, Clinical competence, Midwifery

## Abstract

**Background:**

Vaginal birth management is vital to midwifery education and practice in which midwives are most likely to be directly involved. This situation requires strong cognitive, technical, communicational, and teamwork skills. Therefore, the present study was conducted to evaluate the effect of normal vaginal birth simulation training before formal clinical education on the clinical skills of midwifery students compared with routine clinical education.

**Methods:**

This quasi-experimental study was conducted at the Shoushtar Faculty of Medical Sciences from September 2018 to August 2021. Sixty-one midwifery students participated in the intervention group (n = 31) and in the control group (n = 30). The intervention group participated in the simulation-based training before entering the formal clinical education courses. The control group received no simulation-based training before their formal clinical education. The clinical skills of these students for performing normal vaginal birth in the real field were evaluated by observational examination in the three years (fourth, fifth, and sixth semesters). Data were analyzed by descriptive (mean, SD, and percentage) and inferential statistics (independent t-test and chi-square). A P-value less than 0.05 was considered significant.

**Results:**

The mean score of midwives’ skills in the control group was (28.10 ± 3.42) and in the intervention group, it was (31.15 ± 4.30). The difference in the skill score between the groups was statistically significant (3.40 ± 0.68). The results showed that in the intervention group, 29 students (93.93%) were evaluated from a good to an excellent level, while only ten students (32.71%) in the control group achieved a good level, and others (n = 30) were evaluated at a low level (p < .001).

**Conclusion:**

The results of the present study indicated that the simulation situation for critical skills, such as vaginal birth skills, was significantly more effective than workplace-based learning situations.

**Supplementary Information:**

The online version contains supplementary material available at 10.1186/s12909-023-04319-9.

## Introduction

Over the past two decades, tremendous growth has been observed in undergraduate clinical education models to promote active and self-directed learning, which has developed a method for rapidly incorporating new educational technologies. These models include experiential, exploratory, problem-based, and task-based learning [1]. The Association of Professors of Gynecology and Obstetrics has recommended these learning strategies to augment the clinical experiences and skill acquisition because traditional learning utilizing laboring patients “may lead to poor or incomplete skill acquisition in a fast-paced, high-stress learning environment without standardization of knowledge expectations.” [2]. It is believed that simulation-based learning is a promising approach to meet the Association of Professors of Obstetrics and Gynecology recommendations because it accommodates diverse learning styles and allows for better integration of theoretical and practical concepts [3]. Simulation-based training allows trainees to practice in a safe and protected environment, practice more, provide the opportunity for group training, and participate in various simulated experiences [4]. The benefits of teaching through simulation have been emphasized by leading clinical training institutions not only to overcome emerging problems, such as increasing demand for nurses and the need to challenge patients’ rights but also to bridge the gap between theory and practice [5]. Another study revealed that training in a simulated environment promotes academic motivation, nursing skills, self-confidence, and satisfaction among nursing students [6]. Similar study also reported that simulation-based training was superior to other teaching methods and statistically improved self-efficacy in pre-test and post-test studies [7].

On the other hand, in a study comparing simulation and different traditional methodologies, no clear difference in the achievement of learning objectives was evidenced. It was then reported that simulation-based education could not replace other teaching-learning strategies but could improve existing learning methods [8]. Normal birth management is a key part of midwifery education and practice in which midwives are most likely to be directly involved. Properly managing this situation requires strong cognitive, technical, communicational, and teamwork skills [9]. Performing an uncomplicated normal vaginal birth is an essential skill to be learned by undergraduate midwifery students. The International Confederation of Midwives (ICM) has developed a set of qualifications for the initial assessment of midwifery students in which managing a safe spontaneous vaginal delivery is among these conditions [10]. The results of several studies have shown that using simulation models for normal vaginal birth management training can effectively reduce infant brachial plexus injuries, birth trauma, emergency cesarean birth, infant mortality, and postpartum hemorrhage [11]. Also, the clinical trial results revealed that students participating in normal vaginal delivery simulation workshops have more self-confidence and knowledge in performing normal vaginal delivery [12]. In this regard, the studies that used simulation in Obstetrics and Gynecology were reviewed, and it was found that few studies focus on undergraduate students. These studies assessed the effect of mannequin simulation on the cognitive skills of midwifery students as a primary outcome [13].

Teachers help students work independently, engage in decision-making, and improve their skills for normal vaginal delivery. However, various studies showed that the students in the internship course have not been able to improve their professional capabilities due to the challenges [14–16]. The challenges of clinical education include the lack of clear task descriptions for students and instructors, the lack of appropriateness and coordination between the received materials and their application in the hospital, the lack of comfort and educational facilities, and the decline in the academic level of students [14–16].

## Methods

This quasi-experimental study was conducted on midwifery students from September 2018 to August 2021 at Shoushtar Faculty of Medical Sciences, southwest Iran.

### Participant

: The midwifery students participated in the study for three years (the fourth, fifth, and sixth academic semesters). The inclusion criteria of participants included a willingness to participate in the study and not passing the previous courses on vaginal birth. The participants had working experiences related to childbirth. Students with recognizable diseases affecting their learning ability (such as hand-eye coordination problems (Dyspraxia) and feeling extreme stress, or having accidents during the training and evaluation sessions were excluded from the study. Having identified the eligible students, all participants who studied in the investigated school entered the study. The participants were randomly divided into two groups: intervention and control. The students were assigned to the intervention and group based on systemic randomization. In order to do this, we calculated and fixed the sampling interval. After that, we selected a random starting point and repeated the sampling interval to select subsequent elements.

### Instructor and evaluators

- A trained instructor (MSc in midwifery) with ten years of working experience in midwifery facilitated the students’ learning process in the intervention and control groups. They are faculty members at the Shoushtar Faculty of Medical Sciences.
- Two evaluators with an MSc in midwifery with ten years of working experience evaluated the student’s performance in both groups. They were female, and the mean age of the instructor and evaluators in this study was 38 years. They all had a valid midwifery certificate from an Iranian medical science university from the first-class Ministry of health in Iran.

#### Educational intervention

The participants in the intervention group attended simulation-based training before formal clinical education. All students were female.

- The main objective of the training for the intervention group was to help perform an uncomplicated cephalic birth. The educational content of the workshop is shown in Table 1. The students in the intervention group participated in a three-day workshop to exercise the process of cephalic birth on a pelvic and vaginal birth moulage made by M and Nasco companies. On the first day of the workshop, the instructor explained the workshop’s objectives, the course’s educational content, and the sessions’ schedule to the students. Then, in one hour, she gave explanations in the form of lectures, slide shows, and videos about the vaginal examination, pelvic examination, and how to give birth with a cephalic presentation, and then, she practically showed them how to give birth on the moulage to the students.
- Afterward, each student was asked to practice normal vaginal delivery on the moulage. The skills were individually taught, and the students repeated these independently. The formative assessment of the students was conducted by evaluating the student’s performance of simulated births (at least 3) under the instructor’s supervision. The instructor provided constructive feedback. In the next sessions, the instructor addressed the practical training of the students. In each meeting, enough time was allocated to answer the questions and solve the students’ skill problems.
- Subsequently, the students experienced formal clinical education in the hospital’s maternity unit that used lectures and practical work in the clinical field. Practical activities of the students in the clinical field included clinical history taking, checking vital signs, Leopold’s maneuver, fetal heart rate checking, vaginal examination, taking IV line, sending the necessary tests, checking the course of birth, drawing a partograph, and giving birth under the supervision of the instructor. In this step, the students exercise the process of cephalic birth in supervising the instructors. The students could involve in the process of cephalic birth independently. Each student in the clinical field must independently control at least three pregnant women from labor entry to birth and submit a performance report to the instructor. (A formative assessment in the real situation). During their training, the students were given constructive feedback in the formative assessment process.

**Table 1:** The educational content of the workshop

1. The vaginal examination (dilatation, distension)
2. Pelvic examination (fontanelles and ischial spines)
3. Identification of cephalic presentation and fetal head position, and crowning
4. Delivery with the cephalic presentation, including rotation and descent surgery, protection of the perineum, head removal, neck umbilical cord check, shoulder and trunk removal, placing the baby on the mother’s chest for skin-to-skin contact, assessment of placenta separation, controlled umbilical cord tension, placenta birth, examination and monitoring of uterus contraction and umbilical cord clamping after childbirth

#### Control Group

In the control group, the students participated in formal clinical education that used lectures and practical work in the clinical field. They have no simulation-based training before the formal courses. The practical actions of the students in the control groups were similar to the intervention group. Likewise, the intervention group, each student must independently control at least three pregnant women from labor entry to birth in the clinical field and submit a performance report to the instructor. During their training, the instructors delivered constructive feedback in the formative assessment process in the clinical field (A formative assessment in a real situation).

#### Summative assessment process

The clinical skill of normal vaginal birth among the students in the intervention and control group was assessed by observational examination in a real situation. Two trained evaluators assessed the students using a checklist in the labor room. The evaluators were educated on the observational examination principles, the checklist items, and scoring in a 2-hour session. Additionally, the evaluators were asked to practice the evaluation process of students from labor entry to delivery in a simulated situation.

The blinded evaluators were assigned to the groups to assess the students during their clinical placement when caring for real women during labor and birth. The evaluators assessed the student’s skills in the process of a real-life patient birth in a labor situation.

To investigate the long-term effects of the training course, we evaluated the same students over three years (the fourth, fifth, and sixth academic semesters).

#### Measures

The students’ clinical skills were assessed by an 8-question checklist based on a Likert scale (unacceptable, very poor, poor, average, good, and excellent). The range of scores of this checklist was between 8 and 48 points, and higher scores indicated higher levels of students’ skills in childbirth. The content validity of the checklist was approved by ten midwifery faculty members. This checklist was finalized after going through two stages by applying experts’ opinions. The pilot test was done for ten students to assess the reliability of the checklist items using internal consistency (Cronbach’s alpha = 0.822). A related checklist examined skills such as head control, rotation, umbilical cord checking, head removal, body removal, grasping the baby, and skin-to-skin contact.

#### Data analysis

The Shapiro-Wilks test was used to check the normality of data distribution. Since the data were normally distributed, we used the independent t-test to compare the two study groups, the ANOVA test, and the Generalized Estimating Equations model to compare the students’ performance in the three semesters. Data analysis was done using SPSS version 21 software. The significance level of the above tests was considered P-value < 0.05.

### Ethical consideration

The current study was approved by the ethics committee at the Shoushtar Faculty of Medical Sciences (IR.SHOUSHTAR.REC.1397.007) in the southwest of Iran (Shoushtar). The study’s objectives were explained to all students, and it was emphasized that participation was completely voluntary and did not interfere with the subsequent educational program or with final grades. All students completed the written informed consent to participate in the study.

## Results

In this study, 61 Shoushtar Faculty of Medical Sciences students participated; 31 (50.81%) were placed in the intervention group and 30 (49.18%) in the control group. The mean age of the students was 21.03 years ± 0.98 with a range (of 19–25). Table 2 shows students’ mean scores of normal vaginal birth skills in the fourth, fifth, and sixth semesters. In the 4th semester of study, there was a statistical difference between the intervention and control groups in the three skills of controlling the birthing of the head, the manner of birthing body, and umbilical cord clamp (p < .001, p = .006, p = .046), but in other skills, no significant difference was observed between the items measured (p > .05) (Table 2).

**Table 2:** Comparing the students’ scores of normal vaginal delivery skills

	4th semester				5th semester				6th semester			
Delivery skill questions	Intervention group	Control group	Difference of means	p-value*	Intervention group	Control group	Difference of means	p-value*	Intervention group	Control group	Difference of means	p-value*
1-Did the student perform the control of the perineum when exiting the birth canal correctly?	3.21 ± 0.42	2.66 ± 0.48	0.55 ± 0.13	0.000	3.81 ± 0.73	3.36 ± 0.49	0.45 ± 0.18	0.020	3.90 ± 0.70	3.68 ± 0.64	0.22 ± 0.20	0.284
2- Did the student turn the head correctly?	3.21 ± 0.42	2.66 ± 0.48	0.55 ± 0.13	0.000	3.63 ± 0.65	3.18 ± 0.39	0.45 ± 0.16	0.008	4.04 ± 0.66	3.54 ± 0.59	0.50 ± 0.19	0.013
3- Did the student check the umbilical cord around the neck correctly?	3.21 ± 0.51	3.00 ± 0.44	0.21 ± 0.14	0.146	3.81 ± 0.66	3.31 ± 0.47	0.50 ± 0.17	0.006	4.57 ± 0.50	4.00 ± 0.53	0.57 ± 0.15	0.001
4- Did the student hold and removed the baby’s head correctly?	3.39 ± 0.49	3.19 ± 0.40	0.20 ± 0.13	0.152	4.36 ± 0.65	3.54 ± 0.67	0.81 ± 0.20	0.000	4.19 ± 0.60	3.68 ± 0.47	0.50 ± 0.16	0.004
5- Did the student deliver the baby’s body correctly?	3.26 ± 0.44	3.09 ± 0.30	0.16 ± 0.11	0.162	4.27 ± 0.82	3.50 ± 0.74	0.77 ± 0.23	0.002	4.52 ± 0.67	4.13 ± 0.63	0.38 ± 0.20	0.061
6- Has the student done the filling correctly?	3.39 ± 0.49	3.04 ± 0.21	0.34 ± 0.11	0.006	4.50 ± 0.74	4.00 ± 0.75	0.50 ± 0.22	0.032	4.71 ± 0.84	4.27 ± 0.76	0.44 ± 0.24	0.080
7- Did the student carry the baby correctly?	3.30 ± 0.55	3.19 ± 0.51	0.11 ± 0.16	0.486	4.22 ± 0.92	3.59 ± 0.66	0.63 ± 0.24	0.012	4.85 ± 0.79	4.27 ± 0.45	0.58 ± 0.19	0.005
8- Did the student clamp the umbilical cord correctly?	3.21 ± 0.42	2.95 ± 0.58	0.26 ± 0.15	0.092	4.31 ± 0.83	3.72 ± 0.63	0.59 ± 0.22	0.012	4.71 ± 0.71	4.04 ± 0.37	0.66 ± 0.17	0.000
Mean skill scores	3.47 ± 0.51	3.14 ± 0.57	0.33 ± 0.16	0.046	4.11 ± 0.23	3.52 ± 0.20	0.59 ± 0.06	0.000	4.44 ± 0.24	3.95 ± 0.21	0.48 ± 0.07	0.000

**Fig. 1:**
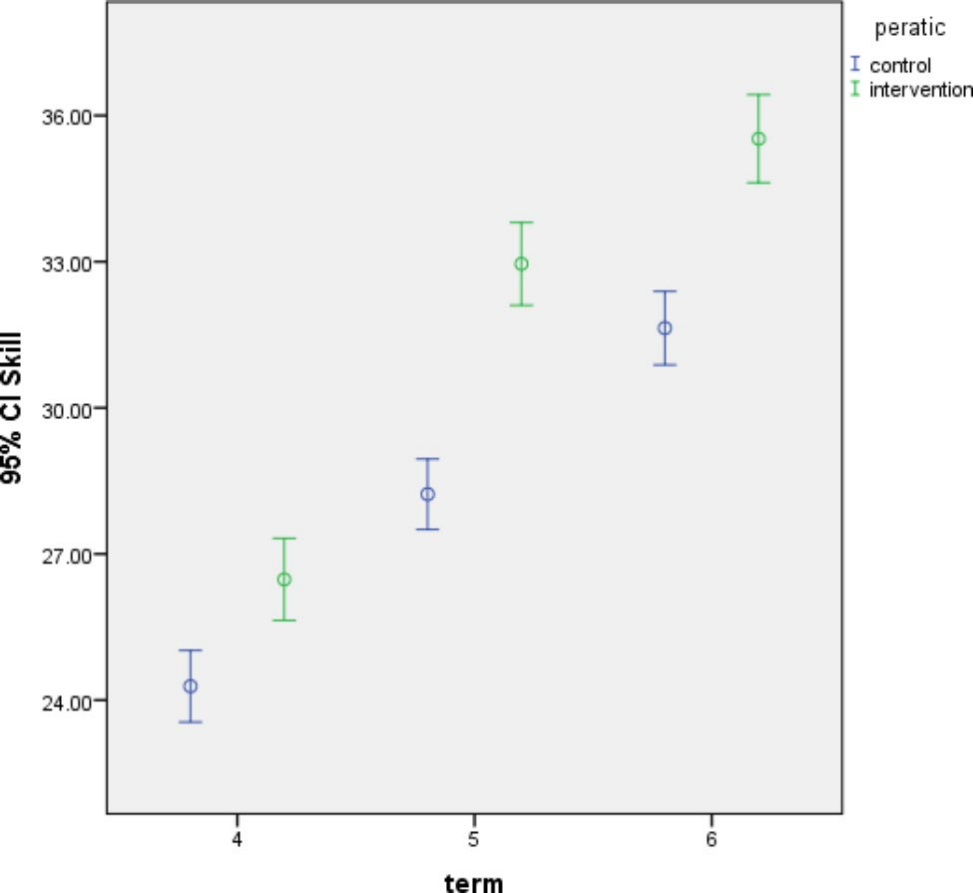
Comparison of students’ skills in three academic semesters by control and intervention group

There was a significant difference between their scores in the two intervention and control groups (p < .001). Also, there is a statistically significant difference between the control and intervention groups in the mean normal vaginal birth skills score in 3 semesters (p < .001). The total score of childbirth skills in the control group was 28.10 ± 3.42; in the intervention group, it was 31.15 ± 4.30 (Table 2). Besides, the difference in the skill score between the groups was calculated (3.40 ± 0.68), which was statistically significant (p < .001) (Table 2; Fig. 1).

The results listed in Table 3 show that, based on the Generalized Estimating Equations (GEE) method, students’ skill scores have changed in general in different periods (time-variable) (p < .001) and also under the influence of different investigated groups (p < .001) and also, the studied groups in different periods (interaction effect of group * time) had an effect on the changes occurred in the students’ skills at the 95% confidence level (p < .001) (Table 4).

**Table 3:** Comparing the mean and standard deviation of students’ scores from the normal vaginal delivery skills questionnaire in a total of 3 semesters

Delivery skill questions	Intervention group (n = 31)	Control group (n = 30)	Difference of means	p-value*
1- Was the control of the perineum when exiting the birth canal performed correctly?	3.63 ± 0.69	3.24 ± 0.68	0.39 ± 0.12	0.002
2- Did the student turn the head correctly?	3.62 ± 0.69	3.24 ± 0.53	0.37 ± 0.10	0.001
3- Did the student check the umbilical cord around the neck correctly?	3.90 ± 0.73	3.50 ± 0.58	0.40 ± 0.11	0.001
4- Did the student hold and removed the baby’s head correctly?	3.92 ± 0.75	3.44 ± 0.55	0.47 ± 0.11	0.000
5- Did the student deliver the baby’s body correctly?	4.04 ± 0.83	3.56 ± 0.72	0.47 ± 0.13	0.001
6- Has the student done the filling correctly?	4.15 ± 0.94	3.83 ± 0.82	0.32 ± 0.15	0.041
7- Did the student carry the baby correctly?	4.07 ± 0.99	3.61 ± 0.78	0.46 ± 0.15	0.004
8- Did the student clamp the umbilical cord correctly?	4.15 ± 0.86	3.64 ± 0.64	0.50 ± 0.13	0.000
Average total skill scores	3.93 ± 0.53	3.51 ± 0.42	0.42 ± 0.08	0.000
p-value**				

**Table 4:** Examining the effect of treatment groups, different times and the effect of group*time, for students’ birth skills *

Variable	effects	Wald Chi-Square	p-value	Partial Eta Squared
Skill	Group	230.94	0.000	0.509
	Time	374.57	0.000	0.786
	Group * Time	28.49	0.000	0.082

The findings of the study showed that 29 (93.93) of the students in the intervention group were at a good and excellent skill level, and only 10 (32.71%) of the students in the control group were at a good skill level and the rest was at lower levels (Table 5).

**Table 5:** The frequency of scores obtained by students from the skill questionnaire by the control and intervention groups by semester

Semester	Control(n = 30)					Intervention(n = 31)					
	Very weak	weak	medium	Good	Excellent	Very weak	weak	medium	Good	Excellent	
4	1(3.33)	1(3.33)	26(86.66)	2(6.66)	0(0.0)	0(0.0)	1(3.22)	14(45.16)	16(51.61)	0(0.0)	
5	0(0.0)	1(3.33)	9(30.0)	20(66.66)	0(0.0)	0(0.0)	0(0.0)	3(9.67)	15(48.38)	33(16.66)	
6	0(0.0)	0(0.0)	2(6.66)	28(93.33)	0(0.0)	0(0.0)	0(0.0)	1(3.22)	11(35.48)	135(68.2)	
Total	1(3.33)	1(3.33)	18(60.00)	10(33.33)	0(0.0)	0(0.0)	0(0.0)	2(6.45)	25(80.64)	26(13.13)	

## Discussion

The present study showed that simulation-based training as a preparation course before formal clinical education is associated with improving the students’ skills in normal vaginal delivery, and this effect was long-lasting.

The simulation training increased the efficiency of the learning process by providing the opportunity to practice and repeat skills in a safe environment [14]. The normal vaginal delivery process can become a complicated emergency, especially when not performed safely, leading to unpredictable maternal morbidity and mortality [17]. The limitations of bedside learning and the challenges of patient rights lead to innovative educational methods such as simulation [18]. Simulation-based training of clinical skills in a university can meet students’ educational needs, progress, and patient rights. There are many differences between clinical skills training centers around the world because of the type of training chart, the number of facilities, the field of activity, the target group, and the training resources. However, each aims to train, share clinical practice information, and improve patient interaction [19]. A simulation-based training improves knowledge and skills. Simulation-based training is suitable for teaching emergency obstetrics and newborn care since it allows for feedback, practice, and trial and error[20]. Simulation is an expensive training method, especially when used to teach skills, and robust research is important to justify its routine use and cost [21].

In our study, the simulation–based training before formal clinical education assisted the student to achieve preparation of understanding of the steps and coping with the stress. These experiences help the student to manage new situations in real opportunities. In addition, they could retrieve their learning and experience to respond to the new activities in real situations. Simulation training was found to increase normal vaginal birth skills, which is consistent with the findings of a the present study [15]. A systematic review discovered that workers’ ability to perform emergency services and normal vaginal birth care was improved with simulation-based training. [16]. There is growing evidence that clinical skills acquired in medical simulation environments improve, patient care practices and patient outcomes. In gynecology and obstetrics emergency practice, simulation has been used to improve patient care practices, including management of shoulder dystocia, third-stage hemorrhage, umbilical cord Prolapse, eclampsia, breech birth, and cesarean Sects. [22–24]. Also, in a similar study, the care skills of midwifery and nursing students were increased after using simulation [25]. Studies showed that simulation training increased self-efficacy and perineal repair skills among participants [26, 27]. Moreover, the effectiveness of ambulance simulation training in improving first aid and emergency care skills was demonstrated in a different study [28].

### Limitations

Participants in this study had no prior experience in birth departments or with obstetrics and gynecology simulators. Additionally, randomization ensured similar distribution between groups. Nevertheless, other unmeasured differences in group characteristics may have influenced the results. Simulation-based learning was also limited by the fact that the practice environment for simulation training could not provide a large group for individual training. This study also had several strengths, including having a control group that was compared with the intervention group. The learned skills in the simulated environment were evaluated on real patients in the clinical arena in three consecutive semesters by two separate instructors. In addition, the lack of student surveys of pre and post-knowledge/confidence acquisition was a limitation of the present study.

## Conclusion

Normal vaginal birth simulation training increased students’ performance of normal vaginal birth in a real environment during the time. According to these results, it is recommended to use normal vaginal birth simulation in the midwifery curriculum.

## Electronic supplementary material

Below is the link to the electronic supplementary material.

Supplementary Material 1

## Data Availability

The datasets used and/or analyzed during the current study are available from the corresponding author on reasonable request. The data are not publically available, because of the psedonymisation and data protection guidelines according to the ethics approval.
