# Supplementary material for: The effects of normal vaginal birth simulation training on the clinical skills of midwifery students: a quasi-experiment study
Source: BMC Med Educ. 2023 May 19;23:353. doi: 10.1186/s12909-023-04319-9 (PMC10199639; doi:10.1186/s12909-023-04319-9)
Supplement: Supplementary file 1 — Supplementary Material 1 [file 12909_2023_4319_MOESM1_ESM.doc]

**The Effects of Normal Vaginal Birth Simulation Training on the Clinical Skills of Midwifery Students: A Quasi-Experiment Study**

**Running Title:** simulation training of normal vaginal birth

### Zahra Sadat Pajohideh, ^1^ Solmaz Mohammadi^2^, Fatemeh Keshmiri ^3^, Azam Jahangirimehr ^4^, Azam Honarmandpour^1*^

1. MSc of Midwifery, Department of Midwifery, Shoushtar Faculty of Medical Sciences, Shoushtar, Iran.

2.Reproductive Health Promotion Research Center, Ahvaz Jundishapur University of Medical Sciences, Ahvaz, Iran.

3. Medical Education Department, Educational Developmental Center, Shahid Sadoughi University of Medical Sciences, Yazd, Iran.

4. MSc of Biostatistics, Shoushtar Faculty of Medical Sciences, Shoushtar, Iran.

**Clear massage:** From the obtained results it could be concluded that, Normal vaginal delivery simulation training increased students' self-efficacy in performing natural childbirth in a real environment. According to these results, it is recommended to include natural childbirth simulation in the preclinical practice of the midwifery curriculum.

**Number of abstract words:** 248 **words**

**Total number of words**: 3127 words

**Number of tables:** 6 tables

**Number of figures:** 1 figure

**Article Type:** original research

**Conflict of Interest:** The authors declare that they have no conflict of interest.

**Financial Disclosure:** The authors declare that no competing financial interests exist.

**Funding/Support:** This study was supported by Shoushtar Faculty of Medical Sciences, Shoushtar, Iran.

**Ethical code:** IR.SHOUSHTAR.REC.1397.007).

**Authors’ Contribution:**

ZP, SM, FK, AJ and AH led the overall development of the study (Conceptualization, Methodology, Validation, and Investigation). AH, and SM led the development of the manuscript (the first and original draft). AH led the supervision of the research implementation. AH, and ZP contributed to the development of the data collection methods and conduct of the data collection. AJ led the data analysis. All authors reviewed drafts of the manuscript and approved the final version. Corresponding author AH had full access to all of the data in this study and takes complete responsibility for the integrity of the data and the accuracy of the data analysis.”

**Acknowledgement**

We thank all the participants who volunteered for this study; this study is derived from a research project Shoushtar faculty of Medical Sciences (Ethical code: IR.SHOUSHTAR.REC.1397.007) in the southwest of Iran (Shoushtar). All expenses of this study were provided by Shoushtar Faculty of Medical Sciences.

**Transparency statement**

"The Corresponding author Azam Honarmandpour affirms that this manuscript is an honest, accurate, and transparent account of the study being reported; that no important aspects of the study have been omitted; and that any discrepancies from the study as planned (and, if relevant, registered) have been explained".

**Data Availability Statement**

The authors confirm that the data supporting the findings of this study are available within the article and its supplementary materials.

**Corresponding author:** Azam Honarmandpour

**E-mail:** [honarmandpour-a@shoushtarums.ac.ir](mailto:honarmandpour-a@shoushtarums.ac.ir)

**G-mail:** [**honarmandpour.a@gmail.com**](mailto:honarmandpour.a@gmail.com)

‏

**Address:** Rajai Street West. Shoushtar Faculty of Medical Sciences, Shoushtar, Khuzestan, Iran.

**Tel:**  +989166224524

**Fax:** +9806136222323

**Postal code**: 84534-64516

**Email address of authors:**

Fatemeh Keshmiri **G-mail: keshmiri1395@gmail.com**

Zahra Sadat Pajohideh **G-mail: pajohidehz@gmail.com**

solmaz Mohammadi **G-mail: sl.mohammadi89@yahoo.com**

Azam Jahangirimehr **G-mail: a.jahangirimehr@gmail.com**

**Orcid of authors:**

Orcid of Azam Honarmandpour: 0000-0001-6264-2533

Orcid of Zahra Sadat Pajohideh: [0000-0001-6714-](https://orcid.org/0000-0002-3171-479X)2335

Orcid of Azam Jahangirimehr: 0000-0001-6044-2175

Orcid of solmaz Mohammadi: 0000-0002-8124-8600

Orcid of Fatemeh Keshmiri: 0000-0002-6791-2579
